# Down syndrome, accelerated aging and immunosenescence

**DOI:** 10.1007/s00281-020-00804-1

**Published:** 2020-07-23

**Authors:** Noémie Gensous, Maria Giulia Bacalini, Claudio Franceschi, Paolo Garagnani

**Affiliations:** 1 Department of Experimental, Diagnostic and Specialty Medicine, Alma Mater Studiorum, University of Bologna, 40138 Bologna, Italy; 2 ImmunoConcEpT CNRS UMR 5164, University of Bordeaux, Bordeaux, France; 3 Department of Internal Medicine and Clinical Immunology, CHU Bordeaux (Groupe Hospitalier Saint-André), 33076 Bordeaux, France; 4 IRCCS Istituto delle Scienze Neurologiche di Bologna, 40139 Bologna, Italy; 5 Laboratory of Systems Medicine of Healthy Aging and Department of Applied Mathematics, Lobachevsky University, Nizhny Novgorod, 603950 Russia; 6 Department of Laboratory Medicine, Clinical Chemistry, Karolinska Institutet, Karolinska University Hospital, 171 76 Stockholm, Sweden; 7 Applied Biomedical Research Center (CRBA), Policlinico S. Orsola-Malpighi Polyclinic, 40138 Bologna, Italy; 8 CNR Institute of Molecular Genetics “Luigi Luca Cavalli-Sforza,” Unit of Bologna, 40136 Bologna, Italy

**Keywords:** Down syndrome, Accelerated aging, Immunosenescence, Inflammaging

## Abstract

Down syndrome is the most common chromosomal disorder, associated with moderate to severe intellectual disability. While life expectancy of Down syndrome population has greatly increased over the last decades, mortality rates are still high and subjects are facing prematurely a phenomenon of atypical and accelerated aging. The presence of an immune impairment in Down syndrome subjects is suggested for a long time by the existence of an increased incidence of infections, the incomplete efficacy of vaccinations, and a high prevalence of autoimmunity. Immunologic abnormalities have been described since many years in this population, both from a numerical and a functional points of view, and these abnormalities can mirror the ones observed during normal aging. In this review, we summarize our knowledge on immunologic disturbances commonly observed in subjects with Down syndrome, and in innate and adaptive immunity, as well as regarding chronic inflammation. We then discuss the role of accelerated aging in these observed abnormalities and finally review the potential age-associated molecular and cellular mechanisms involved.

## Introduction

Down syndrome (DS) or trisomy 21 is the most common known genetic disorder associated with moderate to severe intellectual disability, occurring in approximately 1 out of every 600–700 live births [1, 2]. DS is related to a chromosomal disorder, corresponding to a total or partial trisomy of the autosomal chromosome 21. Even if during the past decades life expectancy in DS population has greatly increased [3–6], DS individuals have still higher mortality rates as compared with other populations [7, 8]. In addition to signs specifically associated with their congenital syndrome, subjects with DS appear to age differently from the general population, and it is evident that they present symptoms of aging ahead of time, leading to the classical description of DS as a progeroid syndrome and as a model of accelerated aging [9–12].

In this work, we review how the phenomenon of aging impacts the immune system in DS subjects. We firstly describe clinical features of DS individuals from an immune point of view. We then summarize evidence on immunosenescence in DS subjects and how it impacts both innate and adaptive immunity, as well as chronic inflammation. Finally, we discuss the potentially age-associated mechanisms involved.

## Accelerated and atypical aging in Down syndrome

DS has long been considered as a progeroid syndrome, as individuals with trisomy 21 start to age prematurely and present precociously conditions usually characteristic of the geriatric population [9–12]. The aging process in DS subjects not only seems to be premature/accelerated but also appears to be atypical and segmental, as it recapitulates many, but not all, of the classical signs and symptoms of aging [13, 14]. Clinically, DS subjects present signs of early aging affecting particularly the neurological system, with an extremely high prevalence of dementia of Alzheimer’s type [15]. Aging affects also prematurely the dermatological, sensory, endocrine, and musculoskeletal systems, leading to high levels of mortality and multi-morbidity in this population [16].

From a biological point of view, the same trend of accelerated aging has been observed with the use of aging biomarkers [17]. The latest and powerful generations of biomarkers which are based on different approaches have been applied to cohorts of DS adults. Interestingly, these different biomarkers show concordantly accelerated aging in subjects with DS, whether epigenetic clocks [18, 19], metabolic biomarkers such as GlycoAge [20], or predicted brain age [21] are tested.

## Clinical features suggesting an immune impairment in Down syndrome individuals

Significant evidence has accumulated on the existence of an immune dysregulation in DS, existing from the very beginning of the life of DS individuals. Firstly, it has been observed that DS subjects are characterized by a higher susceptibility to bacterial infections as compared with the general population, with especially a higher incidence of recurrent and chronic respiratory tract ones [22–25]. DS individuals have furthermore altered responses to vaccinations with conjugate or toxoid vaccines [26–29]. These suboptimal antibody responses potentially worsen the phenomenon of higher susceptibility to infections. Finally, DS subjects have frequently high levels of autoantibodies. While some of them seem to be not related to an eventual clinical significance [30], the others are associated with a higher incidence of organ-specific autoimmune disorders, affecting especially the endocrine system [31–33].

This clinical picture recapitulates the increased incidence of infections, the failure of vaccination, and the high prevalence of autoantibodies that can be observed in older people of the general population, supporting here also the concept of DS as an accelerated aging syndrome. This clinical presentation is the reflect of important alterations in immune homeostasis in DS with significant perturbations in all branches of the immune system, some of them being common with the ones observed during immunosenescence and that will be detailed in the next sections.

## Immunosenescence and inflammaging in Down syndrome

During aging, the immune system shows marked changes which have been gathered under the term immunosenescence [34–37]. There is a progressive decline in several immune parameters, affecting both innate and acquired immunity, as compared with young healthy subjects. Main hallmarks of immunosenescence are a reduction in the output of naïve T cells by the thymus with an accumulation of memory cells and a consequent reduction in the T cell repertoire and in the ability of these cells to respond to novel antigens (whether infectious or vaccinations). Alongside, aging is characterized by the existence of a chronic, sterile, low-grade inflammation named inflammaging [38]. In the next paragraphs, we will try to parallel the observations made on DS subjects’ immune system and the ones observed during normal aging, focusing on adaptive immunity, innate immunity, and inflammation.

### Adaptive immune system

The main changes in the adaptive immune system during normal aging occur in the T cell compartment as T lymphocytes appear to be dramatically affected, with a decrease in naïve T cells and an increase in memory ones [37, 39–41]. The decline in the maintenance of a sizable naïve T cell compartment leads to a major restriction in T cell diversity with increasing age [42, 43]. This restricted T cell repertoire correlates with the inability to mount efficient immune responses to novel antigens. Additionally, changes in the distribution of memory T cell subpopulations are observed during aging. Immunosenescence is characterized by the accumulation of effector memory T cells (TEM) and T effector memory re-expressing CD45RA cells (TEMRA) (defined as CCR7-CD45RA− and CCR7-CD45RA+ respectively), while there is a decrease in central memory T cell (TCM) compartment (defined as CCR7+CD45RA−). The accumulation of terminally differentiated cells with a senescent phenotype during aging has also been more recently associated with the expression of other surface markers, such as CD57, KLRG1, and PD-1 [44]. KLRG1 is considered as an inhibitor of proliferation, whereas PD-1 is associated with immune exhaustion.

In DS, studies on immune system have been mainly focused on the T cell compartment (Table 1). DS subjects seem to have a clear reduction in the number of circulating CD4+ T cells as compared with age-matched controls [45–51]. Beyond this numerical reduction of CD4+ T cells, an impaired maturation has also been observed [52] and imbalances between T cell subpopulations have been described [23, 45, 49, 53–55]. Like during immunosenescence, a reduction in naïve (CD4+ CD45RA+) lymphocytes has been described in DS, as well as an enrichment in memory T cells and a progressive inversion of the CD4+/CD8+ ratio [45–47, 56]. Higher percentages of Th1 and Th17 lymphocytes have been observed in subjects with DS, while there was no difference in Th2 cell percentages, suggesting a discrete imbalance between pro-inflammatory and anti-inflammatory immune responses [49, 57]. Regarding peripheral regulatory T cells, their proportions are increased, but their function seems to be impaired, as their inhibitory activity is decreased as compared with controls [49, 53, 57, 58]. Functional impairment of other T cell subpopulations has also been described, supported by weaker proliferative responses to specific mitogens at all ages [51, 59–62] and imbalanced cytokine production [58, 60, 63]. However, recently, Schoch et al. stated that despite phenotypical signs of immune exhaustion of lymphocyte subsets (quantified by a higher expression of PD-1), their functionality was normal and preserved, as the subjects were able to mount effector T cell responses with normal functional characteristics [49]. Finally, regarding CD8+ T cells, they appear to also be depleted in naïve subsets, with a higher expression of markers of activation (granzyme B, interferon-gamma, and tumor necrosis factor-alpha) and senescence (KLRG1) [57].

**Table 1:** T lymphocyte compartment in subjects with Down syndrome

Author	Year	Number of subjects	Age	Main result
Burgio et al. [62]	1975	83 DS subjects	Age groups: 0–5 years: 21 subjects	Correlation between PHA responsiveness and age (normal response before 11 years old)
		76 age-matched controls	6–10 years: 8 subjects. 11–15 years: 10 subjects. 16–20 years: 18 subjects. 21–25 years: 18 subjects. 26–30 years: 8 subjects	
Franceschi et al. [59]	1981	28 DS subjects	DS subjects: age range: 6–23 years	No difference in lymphocyte counts
		28 age-matched controls	Controls: age range: 10–27 years	Impairment in the PHA and autologous mixed lymphocyte reaction responses
Karttunen et al. [61]	1984	18 DS subjects	DS subjects: mean age = 20 years	Low response to PHA-induced stimulation in half of the DS subjects. Strong negative correlation between the age and the response. Normal IL-2 production
		Controls (*n* = NA)	Controls: adults, mean age NA	Inversion of CD4+/CD8+ ratio
Lockitch et al. [51]	1987	64 DS subjects	DS subjects: age range: 1–19 years	Lower lymphocytes, T lymphocytes, T helper, and suppressor cell counts
		30 age-matched controls	Controls: age range: 1–19 years	Reduced response to PHA in vitro
Cossarizza et al. [47]	1990	15 DS children	NA	Decrease of the absolute number of circulating lymphocytes and of the absolute number of CD4+ cells
		16 age-matched controls		Increase in the percentages of CD8+ cells
Murphy et al. [54]	1992	13 DS subjects	NA	Lower naïve T cells in peripheral blood
		13 age-matched controls		
Barrena et al. [45]	1993	89 DS subjects	30 children with DS (mean age = 7.9 years) and 59 adults with DS (mean age = 34 years)	Decrease in CD4+CD45RA+ cells. Inversion of CD45RA/CD29 ratio
		59 age-matched controls		
Park et al. [60]	2000	43 DS subjects	DS subjects: mean age = 45 years	Lower PHA-stimulated proliferation and IL-2 production in aged individuals (> 30 years old)
		43 age-matched controls	Controls: NA	
Cocchi et al. [46]	2007	30 DS subjects	Children born from 1999 to 2004	In the five first years of life, progressive decrease in the medium values of lymphocytes and CD4+ levels, with a concomitant increase in CD8+ cells. Lower values of lymphocyte and CD4+ cell counts. Lower CD4+/CD8+ ratio
Guazzarotti et al. [52]	2009	25 DS subjects	DS subjects: mean age = 12.2 years	Reduction of percentages and absolute numbers of CD4+ T cells. Reduction of CD4+/CD8+ ratio
		42 age-matched controls	Controls: mean age = 11.7 years	Reduction of naïve CD4+ and CD8+ T cells. Increase in central memory and terminally differentiated CD4+ lymphocytes
Cetiner et al. [63]	2010	32 DS subjects	DS subjects: mean age = 3.9 years	Higher percentage of CD8+ T cells. Similar percentage of CD4+ T cells and similar CD4+/CD8+ ratio
		32 age-matched controls	Controls: mean age = 4.5 years	
Joshi et al. [48]	2011	12 DS subjects	DS subjects: mean age = 9.4 years	Lower percentages of CD3+ T cells and CD8+ T cells, lower absolute numbers of CD4+ T cells. Lower CD4+/CD8+ ratio
		12 age-matched controls	Controls: mean age = 9.4 years	
Trotta et al. [50]	2011	24 DS subjects	DS subjects: mean age = 38 years	Similar percentages of CD3+ T cells. Higher percentages of CD8+ T cells
		21 age-matched controls	Controls: mean age = 43 years	
Pellegrini et al. [53]	2012	29 DS subjects	DS subjects: mean age = 11.4 years	Decrease in CD4+ T cells. Increase in CD8+ T cells. Decrease of CD4+/CD8+ ratio
		29 age-matched controls	Controls: mean age = 9.3 years	Increase in the number of circulating T_reg_ (CD4+CD25+FoxP3+) cells, with impaired function in vitro
Schoch et al. [49]	2017	40 DS subjects	DS subjects: mean age = 7.4 years	Lower levels of CD4+ T cells, no differences in CD8+ T cells
		51 age-matched controls	Controls: mean age = 8.8 years	Higher Th1/Th2 ratio and higher percentages of regulatory T cells
				Higher expression of the inhibitory receptor PD-1 in all T cell subpopulations
Farroni et al. [55]	2018	12 DS subjects	NA	Reduction of Tfh cells in peripheral blood and tonsils. Impairment of the germinal center reaction
		11 age-matched controls		
Mitwalli et al. [23]	2018	150 DS subjects	DS subjects: mean age = 5.56 years	Reduction in total lymphocyte count. Decrease in CD4+ T cells
		100 age-matched controls	Controls: mean age = 6.11 years	
Waugh et al. [56]	2019	18 DS subjects	DS subjects: median age = 29.5 years	No difference in total CD4+ T cells. Decrease in naïve CD4+ T cells. Enrichment in CD27+ CD4+ memory T cells
		18 age-matched controls	Controls: median age = 28.0 years	
Araya et al. [57]	2019	31 DS subjects	DS subjects: median age (cohort 1) = 26.4 years, median age (cohort 2) = 28.3 years, median age (cohort 3) = 29.6 years	Higher percentage of CD8+ T cells. Decreased frequency of CD4+ T cells. Lower CD4/CD8 ratio. Enrichment in differentiated and senescent CD8+ subsets
		40 age-matched controls	Controls: median age (cohort 1) = 33.1 years, median age (cohort 2) = 27.9 years, median age (cohort 3) = 30.2 years	

*DS*, Down syndrome; *NA*, not available; *PHA*, phytohemagglutinin

The thymus is a key lymphoid organ affected by immunosenescence, as it undergoes major changes during aging with a process of involution and shrinking, and a constant decline in the output of newly generated T lymphocytes [39]. In DS subjects, numerous reports have described thymic impairment, with reduced thymic size, decreased intra-thymic expansion of immature T cells, alterations in thymocyte subpopulations, and inefficient intra-thymic maturation [64–69]. T cell receptor (TCR) rearrangement excision circle (TREC) counts can be used to estimate recent thymic lymphocyte emigrants, a reflection of the newly generated T cells. In DS, numbers of TREC+ peripheral blood cells are significantly lower than those in healthy controls, possibly accelerating the early senescence of the immune system, with a negative correlation between age and the levels of TREC+ cells [48, 58, 69].

Aging also affects the humoral compartment, with reduction in B cell numbers and repertoire diversity [70]. In DS, there are lower numbers of circulating B cells as compared with controls [47, 49, 50, 56, 63, 71], with associated changes in B cell subpopulations (Table 2). Thus, defects in memory B cells have been described [29, 48, 71–73] but also decrease in transitional and/or naïve B lymphocytes [56, 71]. DS subjects are characterized by lower serum levels of immunoglobulins IgM, while the other isotypes seem not to be affected [50, 51, 72, 73]. The impairment of the humoral immune system can lead to alterations in the responses to vaccinations [26, 29, 55]. Additionally, it can have an important impact on mucosal immunity, which is fundamental in the digestive and respiratory tracts for protection against infectious diseases, with possible impairment of mucosal IgA secretion rates in DS subjects [74].

**Table 2:** B lymphocyte compartment in subjects with Down syndrome

Author	Year	Number of subjects	Age	Main result
Cossarizza et al. [47]	1990	15 DS children	NA	Decrease of B lymphocytes absolute number and percentage
		16 age-matched controls		
Cetiner et al. [63]	2010	32 DS subjects	DS subjects: mean age = 3.9 years	Lower percentages of B cells
		32 age-matched controls	Controls: mean age = 4.5 years	
Verstegen et al. [71]	2010	95 DS subjects	Children. Mean age NA	Decrease in B lymphocyte counts. Lack of vast expansion of B cells within the first years of life. Decrease in transitional and naive B lymphocytes. Disturbed peripheral B lymphocyte maturation
		33 age-matched controls		
Joshi et al. [48]	2011	12 DS subjects	DS subjects: mean age = 9.4 years	Decrease of B lymphocytes absolute number and percentage. Decreased total memory and class-switched memory B cells
		12 age-matched controls	Controls: mean age = 9.4 years	
Trotta et al. [50]	2011	24 DS subjects	DS subjects: mean age = 38 years	Lower percentages of B lymphocytes
		21 age-matched controls	Controls: mean age = 43 years	
Verstegen et al. [73]	2014	13 DS subjects	DS subjects: mean age = 11.3 years	In blood, decrease in B lymphocytes. Lower numbers of naïve mature B cells. Normal numbers of transitional B cells. Lower numbers of memory B cells
		43 age-matched controls	Controls: mean age = NA	In tonsils, normal germinal center cells
Carsetti et al. [72]	2015	26 DS subjects	DS subjects: median age = 8.74 years	Lower circulating B lymphocytes. Reduction in transitional and mature-naïve B cell numbers. Lower frequency of switched memory B cells specific for vaccine antigens
		26 age-matched controls	Controls: median age = 8 years	
Valentini et al. [29]	2015	15 DS subjects	DS subjects: median age = 6.6 years	Reduction of total, mature, naïve, and transitional B cells. Severe reduction of switched memory B cells
		15 age-matched controls	Controls: median age = 7.8 years	
Schoch et al. [49]	2017	40 DS subjects	DS subjects: mean age= 7.4 years	Lower percentage of B cells
		51 age-matched controls	Controls: mean age = 8.8 years	
Farroni et al. [55]	2018	12 DS subjects	NA	In tonsils, similar frequency of total B cells. Increase in naïve B cells, with reduction in memory B cells
		11 age-matched controls		Impairment of the germinal center reaction
Waugh et al. [56]	2019	18 DS subjects	DS subjects: median age = 29.5 years	Depletion of immature naïve, pre-switched activated and memory B cells. Increase in terminally differentiated plasmablasts. Increase in B cells expressing CD11c (signature phenotype of age-associated B cells)
		18 age-matched controls	Controls: median age = 28.0 years	

*DS*, Down syndrome; *NA*, not available

### Innate immune system

There are fewer reports evaluating the innate immune system in DS, as compared with the work done on the adaptive one.

Regarding the myeloid compartment of the innate immune system, one work has suggested that DS subjects have lower granulocyte counts, lower numbers of myeloid dendritic cells, and higher amounts of pro-inflammatory monocytes (CD14^dim^CD16^+^) in peripheral blood as compared with controls [75]. This shift toward subsets associated with inflammation with an increase in inflammatory monocytes was further confirmed [56].

During normal aging, there is an increase in the proportions of natural killer (NK) cells [76, 77]. Regarding this compartment, results in DS are contradictory. First reports in DS, published in the early 1990s, testified of the existence of an expansion of cells with NK activity markers (CD16, CD56, CD57) [47, 78], which were however found functionally inefficient with a reduced NK activity [78]. In more recent reports, results were more inconsistent. Higher percentages of NK cells were described in three works [49, 56, 63], associated with an heightened state of activation [56], whereas absolute number was found normal [75] or even lower [79] in DS subjects as compared with controls. According to some authors, discrepancies could be partly attributed to the definitions used to define NK cells that did not fully discriminate NK cells from other subpopulations of T cells in the initial publications (no differentiation between CD3− and CD3+ cells).

### Inflammation

Aging is characterized by a chronic, low-grade, and sterile inflammation, called “inflammaging,” which has been directly associated with several age-related conditions [38, 80, 81]. The subclinical accumulation of pro-inflammatory factors progresses in parallel with immunosenescence (i.e., mainly a decrease in less-differentiated subsets associated with increase in terminally differentiated ones), and together, they form a vicious circle. In DS subjects, features of chronic inflammation are strongly present. Individuals with DS have increased spontaneous circulating levels of pro-inflammatory cytokines, such as tumor necrosis factor (TNF)-alpha, interferon (IFN) gamma, or interleukin (IL)-6 and IL-1β [50, 82–87]. This pro-inflammatory cytokine profile was confirmed in a recent meta-analysis performed by Zhang et al. on 19 studies in DS subjects, in which significantly increased circulating levels of TNF-α, IFNγ, and IL-1β were demonstrated [88].

Recent works have shed light on the presence of a constant activation of the IFN response in individuals with DS [56, 57, 87, 89]. On a transcriptomic side, a consistent overexpression and hyperactivation of the interferon transcriptional response have been observed in different cell types [56, 57, 89], while on a protein side, an increased expression of IFN receptors and hypersensitivity to IFN-alpha were described [56]. The overexpression of IFN receptors (among which four of the six are encoded by genes located on chromosome 21 [90]), and especially type I IFN receptor subunit IFNAR1, is present across the entire immune system. In response to IFN stimulation, immune cells of DS individuals are hypersensitive and present a hyperactivation of cell type-specific downstream signaling cascades [56, 57]. This increased interferon signaling has been linked to major circulating proteomic changes that are indicative of chronic auto-inflammation and which could fit in the scope of other inflammatory conditions (such as systemic lupus erythematosus or rheumatoid arthritis) or type I interferonopathies [87].

The chronic pro-inflammatory state observed in DS subjects is likely to greatly contribute to neurodegeneration. Inflammation is indeed considered as an important contributor to neurodegenerative disorders, such as Alzheimer’s disease, which is highly prevalent in this population [91, 92]. Thus, combined measurement of Aβ40 and Aβ42 plasmatic levels with inflammatory molecules (such as TNF-α and IL-6) can be used as a strong predictor of prospective cognitive deterioration in DS individuals [83].

### Is the immune system in Down syndrome intrinsically deficient or victim of accelerated aging?

As we have seen above, quantitative and qualitative alterations in all the compartments of the immune system in DS subjects have been well documented. The exact etiology of these alterations is however still debated, and the unanswered question is the following: is the immune system in DS primarily deficient or is it a victim of accelerated aging?

On one hand, some observations seem to be in line with the presence of an intrinsic defect [71, 79, 93]. Children with DS lack the vast expansion of T cells in the first years of life, and the lymphopenia observed later could be related to the impaired expansion of these cell lines in infancy. The impaired thymic output could be attributable partly to the altered thymic anatomy and function, present from the fetal stage onwards. To this regard, it should also be kept in mind that children with DS are at higher risk of thymectomy during cardiac surgery performed for congenital heart malformation. The information of the existence of a previous thymus resection is not always present in the studies, even though it has been demonstrated that thymectomy in this population can be associated with lower percentages of peripheral T cells [49].

On the other hand, the observed alterations in adaptive immunity in DS appear reminiscent of immunosenescence, with a phenomenon exacerbated with the age of the subjects [94]. For example, levels of T cells expressing TREC are significantly lower in DS individuals as compared with controls, and these levels correlate with the age of the subjects [58]. Moreover, a progressive decrease in the values of CD4+ T cells is observed in the first years of life of DS subjects [46]. From a functional point of view, lymphocyte responsiveness to phytohemagglutinin stimulation appears to be in the normal range during the first decade of life, but decreases progressively thereafter [61, 62]. Of particular importance is the obvious pro-inflammatory state observed in DS subjects, which is really similar to typical inflammaging observed during normal aging of the general population [56].

There is a likely possibility that both phenomena of intrinsic defect and accelerated aging are combined, like it seems to be the case in the mouse model of DS (Ts65Dn) [95]. In the animals, there is a clear decrease of the thymic output of immature T cells with a decline and involution of thymocyte progenitors, but at the same time, peripheral mature lymphocytes appear prematurely senescent and have a reduced capacity of proliferation after polyclonal stimulation [95]. In humans, this scenario appears possible and should be addressed in future studies, at the same time as some limits of the published works should be addressed too. Thus, one important limit of the work carried in DS immune system so far is the great heterogeneity of the individuals included in the studies regarding their chronological age. Observations published were performed on newborns, infants, children, or adults, and the comparability of these results is thus limited. Additionally, within the DS population itself, individuals are characterized by a large variability in terms of phenotype, with a high level of complexity in terms of co-morbidities, associated treatments, and social and cultural environments, which can all have a direct or indirect impact on the immune system.

## Accelerated immunosenescence in Down syndrome subjects: potential causes

Aging is driven by interconnected cellular and molecular mechanisms, which have been gathered under the terms of hallmarks or pillars of aging [96, 97]. These mechanisms are highly altered in DS subjects [17] and thus could participate in the acceleration of the aging process, especially in the immune system. Firstly, it is clear that there is a premature aging of the stem cell compartment in DS individuals, and especially of hematopoietic stem cells (HSC) with a significant decrease in circulating human stem and progenitor cells and a lack of HSC self-renewal [56, 98–100]. Secondly, alterations of metabolism have been described at different levels, with impairment of mitochondrial function and presence of chronic oxidative stress [101–103]. Thus, increased levels of endogenous oxygen species have been observed in lymphocytes from individuals with DS as compared with controls [104]. Additionally, higher levels of macromolecular damage are present in DS subjects, especially occurring on DNA molecules in lymphocytes [104–108], and an acceleration of telomere loss has been described in DS lymphocytes as well [109]. Finally, epigenetic patterns are modified, with differential blood DNA methylation signatures in DS individuals [110, 111] and age-related changes in the epigenetic machinery [112].

## Conclusions

Individuals with DS display a spectrum of clinical and biological abnormalities, which are compatible with an important immune impairment. These disturbances are likely caused by the co-existence of two phenomena, with an intrinsic defect and precocious aging on one hand, and a clear phenomenon of accelerated aging on the other hand. The immune dysregulation affects key cell types, both in myeloid and lymphoid compartments, and is associated with a chronic state of inflammation. The dysregulation of immune responses and the inflammatory state are likely to greatly contribute to age-related diseases and especially to neurodegeneration which is highly prevalent in this specific population [15]. Owing to the high level of co-morbidities present in DS individuals, there is an important need to develop and test novel therapeutic strategies [17, 56], in order to target and possibly tackle the phenomenon of accelerated aging in this population.
